# Hazardous air pollutants and primary liver cancer in Texas

**DOI:** 10.1371/journal.pone.0185610

**Published:** 2017-10-10

**Authors:** Luca Cicalese, Giuseppe Curcuru, Mauro Montalbano, Ali Shirafkan, Jeremias Georgiadis, Cristiana Rastellini

**Affiliations:** 1 Texas Transplant Center, Department of Surgery, University of Texas Medical Branch, Galveston, Texas, United States of America; 2 Department of Chemical, Management, Informatics and Mechanical Engineering, University of Palermo, Palermo, Italy; 3 Istituto Euro-Mediterraneo di Scienza e Tecnologia I.E.ME.S.T., Palermo, Italy; 4 Center for Biomedical Engineering, University of Texas Medical Branch, Galveston, Texas, United States of America; Chang Gung Memorial Hospital Kaohsiung Branch, TAIWAN

## Abstract

The incidence of hepatocellular carcinoma (HCC), the most common primary liver cancer, is increasing in the US and tripled during the past two decades. The reasons for such phenomenon remain poorly understood. Texas is among continental states with the highest incidence of liver cancer with an annual increment of 5.7%. Established risk factors for HCC include Hepatitis B and C (HBV, HCV) viral infection, alcohol, tobacco and suspected risk factors include obesity and diabetes. While distribution of these risk factors in the state of Texas is similar to the national data and homogeneous, the incidence of HCC in this state is exceptionally higher than the national average and appears to be dishomogeneous in various areas of the state suggesting that other non-recognized risk factors might play a role. No population-based studies are currently available investigating the effect of exposure to Hazardous Air Pollutants (HAPs) as a contributing risk factor for liver cancer. Incidence rate of liver cancer in Texas by counties for the time period between 2002 and 2012 was obtained from the Texas Cancer Registry (TCR). Through Principal Component Analysis (PCA) a subgroup of pollutants, explaining almost all the dataset variability, were identified and used to cluster Texas counties. The analysis generated 4 clusters showing liver cancer rate either higher or lower than national average in association with either high or low levels of HAPs emission in the environment. The study shows that the selected relevant HAPs, 10 among 253 analyzed, produce a significant correlation (P = 0.01–0.05) and some of these have been previously identified as carcinogens. An association between the increased production and consequent exposure to these HAPs and a higher presence of liver cancer in certain counties is suggested. This study provides a new insight on this complex multifactorial disease suggesting that environmental substances might play a role in the etiology of this cancer.

## Introduction

Primary liver cancer is the third cause of cancer death in the world and the seventh in the United States [1]. Approximately 90% of the primary liver cancers in the United States are hepatocellular carcinoma (HCC) while the remaining 10% are intrahepatic cholangiocarcinoma [2].

The known etiologic risk factors for HCC are comprised of non-specific cirrhosis (21%), alcohol induced liver disease (16%), HCV infection (10%) and HBV infection (5%). In addition, obesity and diabetes mellitus type two are being suspected to increase the risk [3].

Geographically, incidence and mortality rates for HCC are not equally distributed in the US. In a recent study Altekruse S.F. et al. reported an incidence rate of 5.9 (95% CI; 5.8–5.9) and mortality rate of 4.3 (95% CI; 4.3–4.3) per 100,000 persons in the US [4]. Texas ranks first in the US with an incidence rate of 11.7 (almost double the national rate 95% CI) and fifth with a mortality rate of 8.3 (95% CI) [5]. (Rates are per 100,000 persons and are age-adjusted to the 2000 U.S. population). Despite HCV is considered one of the major etiologic factors for HCC in the US, previous studies have shown that the prevalence of HCV in Texas and nationally are similar (1.79% vs. 1.8%) [6]

According to the latest statistics of alcohol consumption per capita in the U.S from the National Institute on Alcohol Abuse and Alcoholism (NIAAA), the total national amount of alcohol consumption was 2.26 gallons per capita, while in Texas consumption was reported to be lower, 2.00–2.24 gallons per capita [7, 8]. Likewise, prevalence of adults smoking cigarettes in Texas in 2011 was 19.2%, the 14^th^ highest in the nation, with this rate ranging from 11.8 to 29.0% across all states [9–11]. Moreover, Texas is on the 16^th^ place nationwide in terms of obesity with a prevalence of 30.9%, (95% CI; 29.5–32.3) while the national prevalence is 34.9% [12, 13]. Consequently, the distribution and prevalence of these risk factors does not seem to explain the high incidence of HCC observed in Texas, suggesting the existence of other factors that might increase the risk of developing this tumor.

However, Texas is home to the American petroleum industry. Subsequently the population of this state is exposed to the hazardous products related to these industries such as petrochemical derivatives and other environmental pollutants.

The purpose of this study was to analyze the distribution of the HAPs in the attempt to identify possible clusters of Texan counties that show a similarity in exposure to individual pollutants. Secondly, to study the distribution of the liver cancer in such clusters of counties to identify possible correlation between the production, hence exposure to individual HAPs and the incidence of liver cancer.

## Methods

### Data source and variables

The variables used in this study were the age-adjusted incidence rate of primary liver cancer and the levels of emission of HAPs for each county of the state of Texas. Liver cancer incidence rates per counties were provided by the Texas Cancer Registry (TCR) Cancer Epidemiology and Surveillance Branch, Texas Department of State Health Services for 2002–2012, which is the most recent available data to date. The TCR is the 4th largest cancer registry in the United States. Approximately 240,000 reports of cancer are being sent annually from over 500 hospitals, cancer treatment centers, ambulatory surgery centers, and pathology laboratories located throughout the state. All rates are described per 100,000. Rates are age-adjusted to the 2000 U.S. Standard Population. When the number of cases is 0, a value of 0.0 for the rate is reported while rates per counties are suppressed in the TCR if more than zero but less than 16 cases due to the risk of loss of confidentiality (in counties with low population and few cases patients can be identified).

Air pollutant concentrations for every Texas County were obtained from the 2002 National Scale Air toxic Assessment (NATA) of U.S. Environmental Protection Agency’s (EPA) in tons per year *(tpy)*. NATA is EPA's ongoing comprehensive evaluation of air toxics in the U.S. NATA assessments generally include a four step process including: Compile a national emissions inventory from outdoor sources, Estimate ambient concentrations of air toxics across the United States, Estimate population exposures across the United States and Characterize potential public health risks due to inhalation of air toxics.

### Statistical analysis

Pollutants dataset contains 253 pollutants which concentrations are measured. Several HAPs are emitted only in a few Texan counties while others are emitted in a more ubiquitous distribution but with different concentrations in each county. In order to interpret the variability structure of this dataset, Principal Component Analysis (PCA) was performed. Through this multivariate technique it was possible to select a subgroup of pollutants to explain almost all the dataset variability. Data for primary liver cancer are available for 139 counties. For the remaining counties data are suppressed because the number of cases was less than 16. Despite these counties were excluded from the analysis presented herein, these were also analyzed separately with the assumption than <16 per county (extremely low number of cases) was equivalent to zero. From a methodological point of view, two steps were performed. In the first step, the pollutants dataset variability was studied to select those with greatest contribution to the dataset variability. The second step was clustering counties according to the pollutants concentration. To this purpose a Cluster Analysis (CA) was performed. PCA [14] is an unsupervised method that through the analysis of the correlation structure of a set of original variables (air pollutants) finds hypothetical new variables—defined principal components (PCs)—accounting for the greatest possible variance in a multidimensional data set. PCA finds the most informative or explanatory features hidden in the data without needing a-priori knowledge. It accomplishes this purpose by computing a new smaller set of uncorrelated variables (PCs) that represent the original dataset. The first Principal Component (PC1) is a linear combination of the original variables that accounts for the maximum amount of variability in a single direction. The second component (PC2), orthogonal to the first one, accounts for the maximum of the remaining variance and so on. From a more technical point of view, PCA is based on Eigen analysis of the covariance or correlation matrix. In the PCA model, each original variable (pollutant) has a *loading*. The greater the loading the greater contribution of the variable to a meaningful variation in the data. In the same time, each sample (county) is associated to a *score* along each component which reflects the location of the sample in the model. When two components are enough to represent the great part of data variability, the location of each sample along the two directions can be shown in a plane PC2-PC1.

Cluster Analysis is a method for identifying homogenous groups of objects called clusters. At the beginning of the clustering process, variables (air pollutants) are selected for the clustering process to start. In this study, a hierarchical technique was employed. Clusters are then consecutively formed from objects starting with each object representing an individual cluster. According to some similarity measures, these clusters are then merged. There are various measures to express (dis)similarity between pairs of objects. Here the Euclidian distance was used considering the distance (between each pairs of objects) and the shortest, the more similar the objects. The proposed algorithm to combine the most similar objects is the Ward’s method.

This approach does not combine the two most similar objects successively but those objects whose merger increases the overall within-cluster variance to the smallest possible degree. Since hierarchical methods provide only very limited guidance for choosing the number of clusters, the *Elbow method* has been used. By plotting the within-cluster sum of squares (a measure of the compactness of the cluster) by varying the number of clusters according to the number of clusters, a distinctive break (elbow) can be employed to select the number of clusters [15].

After clustering the Texan counties into homogeneous groups, a Discriminant Analysis (DA) was also performed. DA [16, 17] among groups aims at predicting which group a new case belongs to. In most common applications of discriminant analysis, many variables or predictors are considered in order to determine the ones with a high discrimination power with a step-by-step procedure. In this study, all the selected variables (pollutants) are used for discrimination purposes. Considering that the same data set was used both for estimating the DA model and the classification, an over estimation of the Hit Ratio was expected. To avoid this, the leave one-out cross-validation method was employed. This technique works by omitting each observation one at a time, recalculating the classification function using the remaining data, and then classifying the omitted observation.

## Results

**Fig 1:**
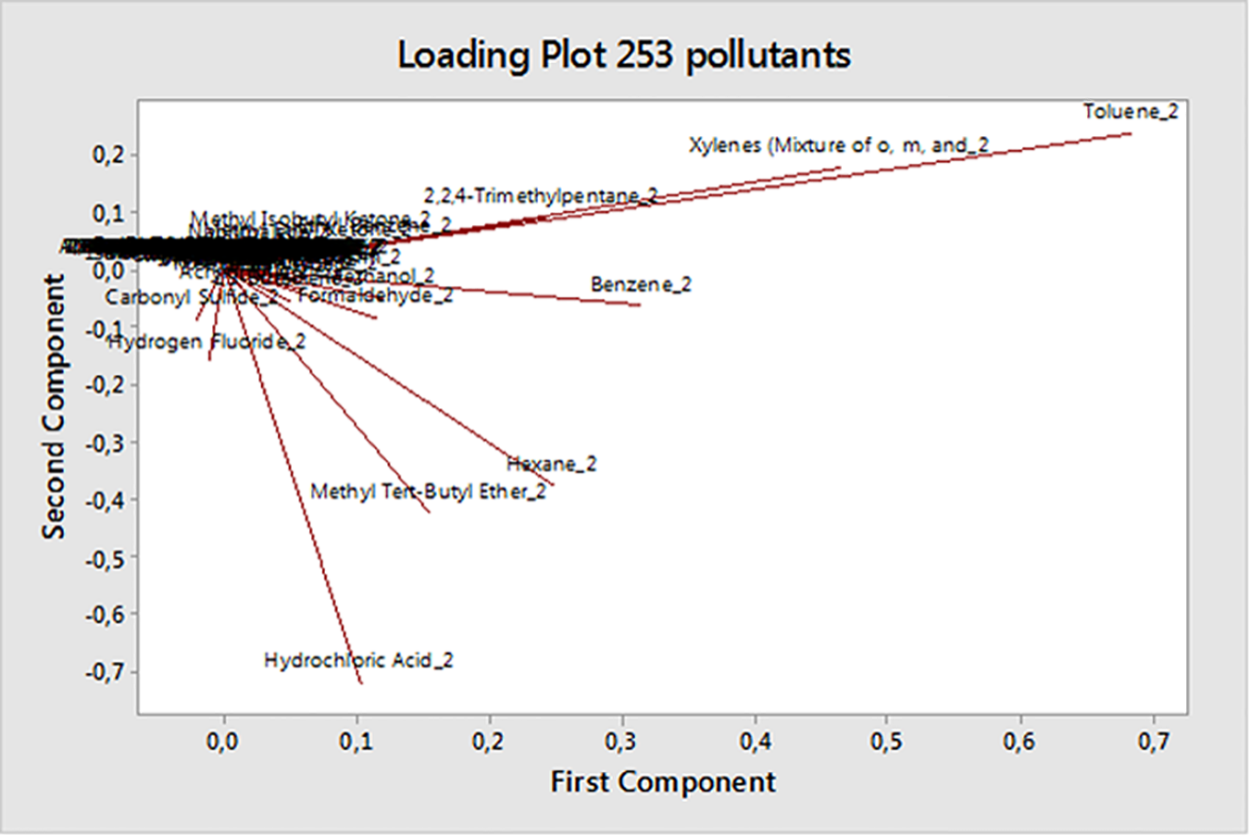
(**A**) Number of principal components and corresponding eigenvalue (%). (**B**) Loading plot for pollutants- PC2 vs PC1.

Two principal components were retained in the PCA for the analysis of pollutants in the Texan counties. The two components account for the 94.9% of the variation (Fig 1A) in the original 253 variables.

Almost all the loadings of the original 253 variables were closed to 0. As shown in Fig 1B, with most pollutants clustered around 0 in the PC2-PC1 plane. However, only 2,2,4-Trimethylpentane, Benzene, Ethyl Benzene, Formaldehyde, Hexane, Hydrochloric Acid, Methanol, Methyl Tert-Butyl Ether, Toluene, Xylenes exhibited distinct loadings. HAPs in PCA analysis have been standardized.

Based on these loadings, the previous group of original variables was selected for further analyses. The 10 selected pollutants contribute mostly to the *scores* of the PCA model, while for those pollutants with loadings closed to 0 the contribution is statistically irrelevant. As shown in Table 1, the first PC was heavily loaded on 2,2,4-Trimethylpentane, Benzene, Ethyl Benzene, Formaldehyde, Methanol, Toluene, Xylenes, while the second PC was heavily loaded on Hexane, Hydrochloric Acid, Methyl Tert-Butyl Ether.

**Table 1:** PC1 and PC2 loadings for the selected pollutants.

Pollutants	PC1	PC2
2,2,4-Trimethylpentane	0,240	0,088
Benzene	0,314	-0,064
Ethyl Benzene	0,114	0,038
Formaldehyde	0,114	-0,084
Hexane	0,247	-0,378
Hydrochloric Acid	0,101	-0,720
Methanol	0,119	-0,051
Methyl Tert-Butyl Ether	0,154	-0,426
Toluene	0,684	0,236
Xylenes	0,464	0,176

The selected pollutants were then employed for the cluster analysis. Counties were grouped accordingly to their similarity in the content of pollutants (Table 2). To this purpose, as mentioned in the methods section, a Euclidean distance as similarity measure and Ward’s method of linkage were adopted. The Elbow Method was used to determine the number of clusters to be considered. Four clusters were identified. In particular, 100 counties belong to cluster 1, 13 to cluster 2, 129 to cluster 3 and 4 to cluster 4.

**Table 2:** Pollutant production (tpy) in each cluster.

MEAN POLLUTANT (*tpy*) PRODUCTION/CLUSTERS				
Pollutants	CLUSTER 1	CLUSTER 2	CLUSTER 3	CLUSTER 4
2.2.4-Trimethylpentane	62.15	253.27	13.98	1150.02
Benzene	89.98	338.88	23.77	1357.42
Ethyl Benzene	33.14	130.23	7.95	568.6
Formaldehyde	59.09	166.11	13.51	733.64
Hexane	52.89	469.51	10.9	1055.55
Hydrochloric Acid	27.84	235.52	1.13	444.51
Methanol	60.06	323.29	10.93	954.83
Methyl Tert-Butyl Ether	21.14	205.64	2.46	2489.3
Toluene	202.63	811.27	47.6	3775.7
Xylenes	146.43	548.15	35.43	2463.04

Stability of the results was assessed by changing the order in the dataset and by re-running the analysis. Results did not change over dataset permutations. Clusters exhibit a high degree of within-segment homogeneity and between-segment heterogeneity. Cluster 1 and 3 exhibit the lowest variance, while cluster 4 the highest one. The latter is constituted by counties reporting the greatest content of pollutants. In order to understand if the four identified segments are distinguishable, clustering variables’ average values of all counties (Table 3) belonging to each cluster were computed and ANOVA was performed. Homoscedasticity and normal distribution for residuals were verified for each variable (pollutant).

**Table 3:** Levene test results for homoscedasticity with four clusters. ANOVA for differences among the four groups.

Pollutants	Levene test (p-value)	ANOVA (p-value)
2.2.4-Trimethylpentane	0.541	<0.0001
Benzene	0.523	<0.0001
Ethyl Benzene	0.704	<0.0001
Formaldehyde	0.108	<0.0001
Hexane	0.126	<0.0001
Hydrochloric Acid	0.000	<0.0001
Methanol	0.082	<0.0001
Methyl Tert-Butyl Ether	0.468	<0.0001
Toluene	0.615	<0.0001
Xylenes	0.580	<0.0001

Levene Test used for homoscedasticity was significant only for Hydrochloric Acid. Thus, for this variable a Welch ANOVA was performed. Residuals were normalized for all the variables. For each variable differences among groups were significant with a common *p-value* less than 0.0001. Post-hoc tests were also performed to know which groups differ. To this purpose Games-Howell Simultaneous Tests were performed for Hydrochloric Acid and Tukey Simultaneous Tests for all the others, For 2.2.4-Trimethylpentane, Benzene, Ethyl Benzene, Toluene, Xylenes and Methyl Tert-Butyl Ether differences of groups are all significant (p<0.0001). As for Formaldehyde, Methanol, Hydrochloric Acid and Hexane results are shown in Table 4.

**Table 4:** Example of post-hoc test for four pollutants in order to show differences for their concentration among clusters.

**Formaldeyde**		**Hexane**	
**Difference of Levels**	**P-value**	**Difference of Levels**	**P-value**
CL2-CL1	<0.0001	CL2-CL1	<0.0001
CL3-CL1	<0.0001	CL3-CL1	<0.0001
CL4-CL1	<0.0001	CL4-CL1	<0.0001
CL3-CL2	<0.0001	CL3-CL2	<0.0001
CL4-CL2	0.002	CL4-CL2	0.092
CL4-CL3	<0.0001	CL4-CL3	<0.0001
**Hydrochloric Acid**		**Methanol**	
**Difference of Levels**	**P-value**	**Difference of Levels**	**P-value**
CL2-CL1	<0.0001	CL2-CL1	<0.0001
CL3-CL1	<0.0001	CL3-CL1	<0.0001
CL4-CL1	0.024	CL4-CL1	<0.0001
CL3-CL2	<0.0001	CL3-CL2	<0.0001
CL4-CL2	0.580	CL4-CL2	0.654
CL4-CL3	0.006	CL4-CL3	<0.0001

Differences between cluster 2 and 4 are not significant for the concentration of Hydrochloric Acid, Methanol and Hexane. Clusters are well distinguishable. Results of the discriminant analysis show that on 246 counties, 220 are correctly classified (89.4%) without cross-validation while, with cross-validation, the correctly classified are 213 (86.6%).

**Fig 2:**
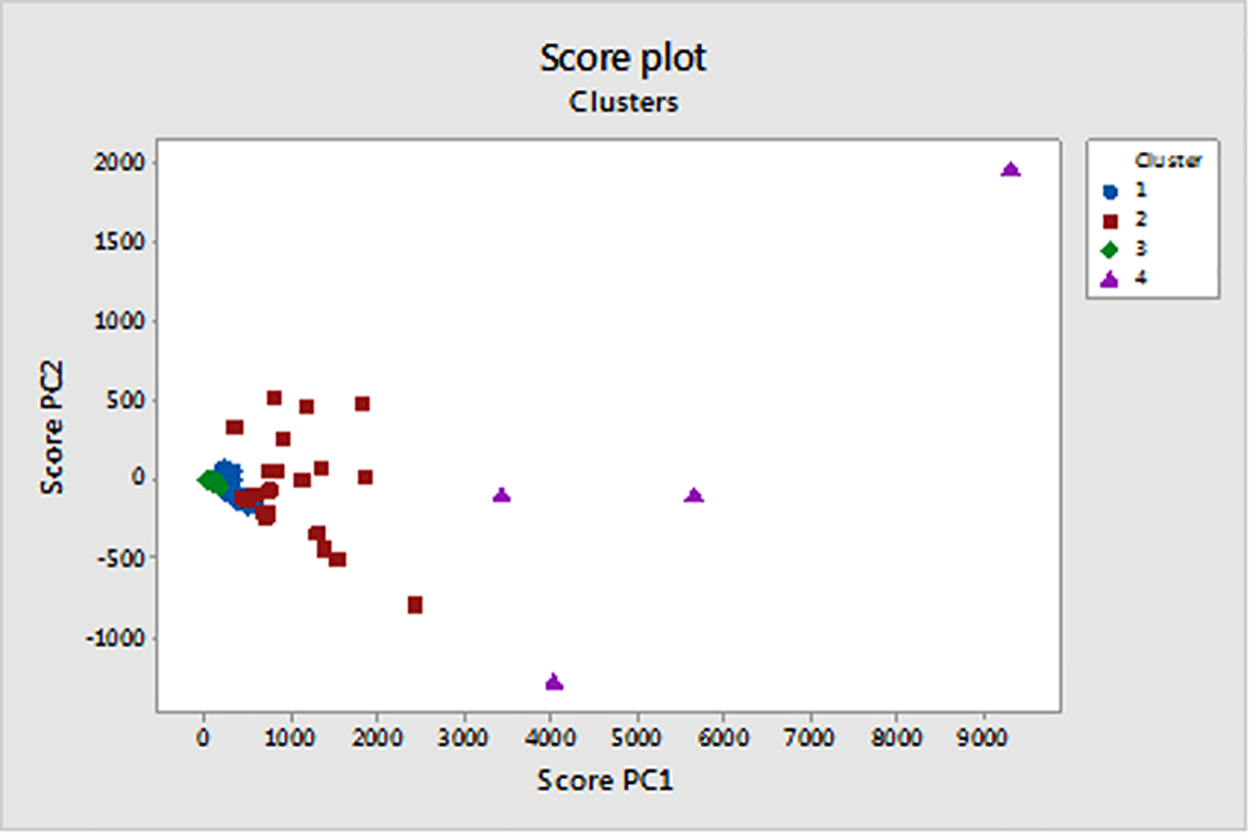
Score plot PC2 vs PC1 with clustering results.

In Fig 2, the counties score plot (plane PC2-PC1) is shown together with the identified clusters. We observed that the first component score is generally greater than the second.

Counties are greater loaded on the PC1 segment of the PCA model where the contribution of 2.2.4-Trimethylpentane, Benzene, Ethyl Benzene, Formaldehyde, Methanol, Toluene and Xylenes is prevalent. In particular, all the counties belonging to Cluster 1 have a PC1 score greater than PC2 (taken in absolute value). The same considerations hold for counties belonging to Cluster 3 and 4. For counties belonging to cluster 2, Grimes County is the only exception to this general behavior. Actually, for the latter PC2 score is greater than the PC1 one. This suggests that generally the first component is the direction where more changes can be observed. To evaluate the prevalence of PC1 scores on PC2 scores, the ratio R between the average PC1 scores and the average PC2 scores for each cluster were calculated. For cluster 1 R is 3.9, for cluster 2 it is 3.8, for cluster 3 and cluster 4 it is 3.5 and 6.4, respectively.

### Liver cancer incidence rates in the counties clusters

**Fig 3:**
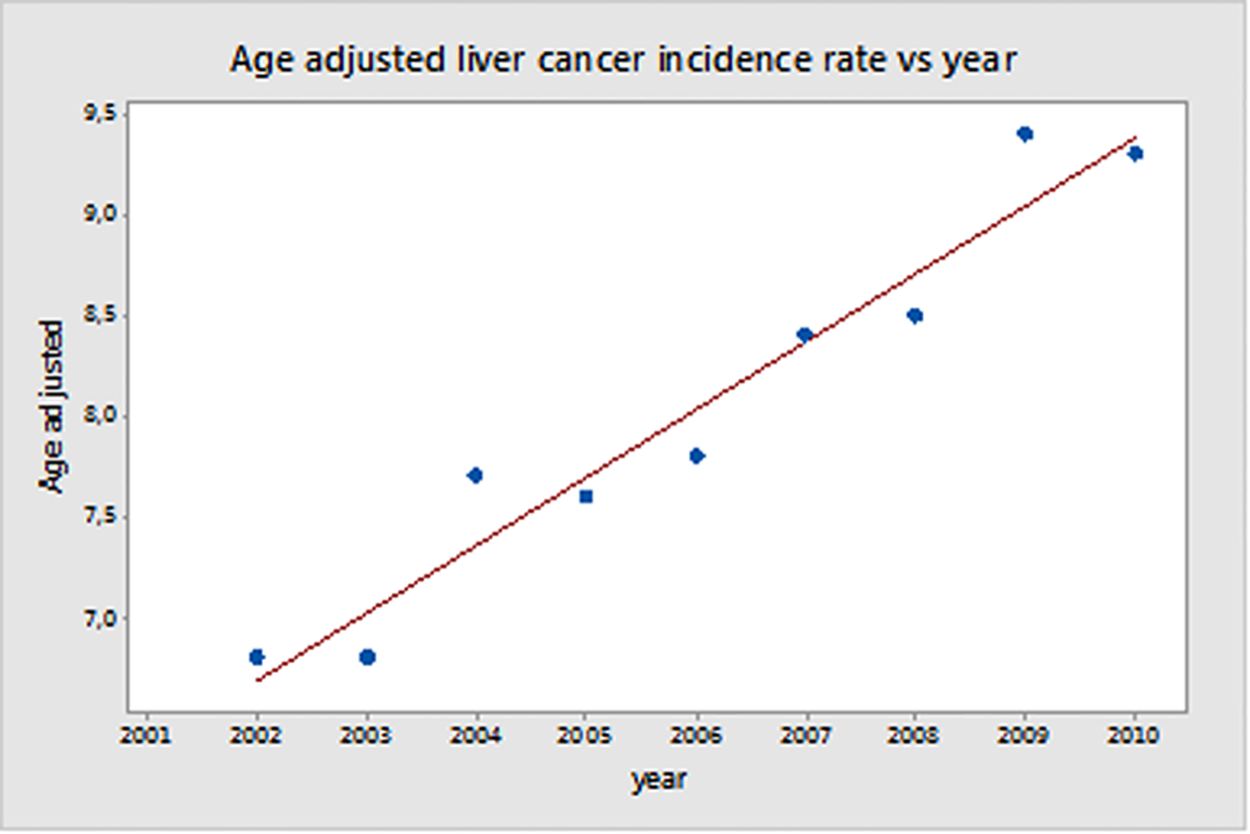
Age-adjusted liver cancer incidence rate for Texas State over time.

An overall increasing incidence of liver cancer was observed in the last ten years in Texas (Texan Cancer Registry), as shown in Fig 3. Primary liver cancer rates by county were taken into account with the aim of detecting a possible “accordance” between the distribution of the environmental pollutants in the identified clusters and the mean incidences rates of cancer per cluster.

**Fig 4:**
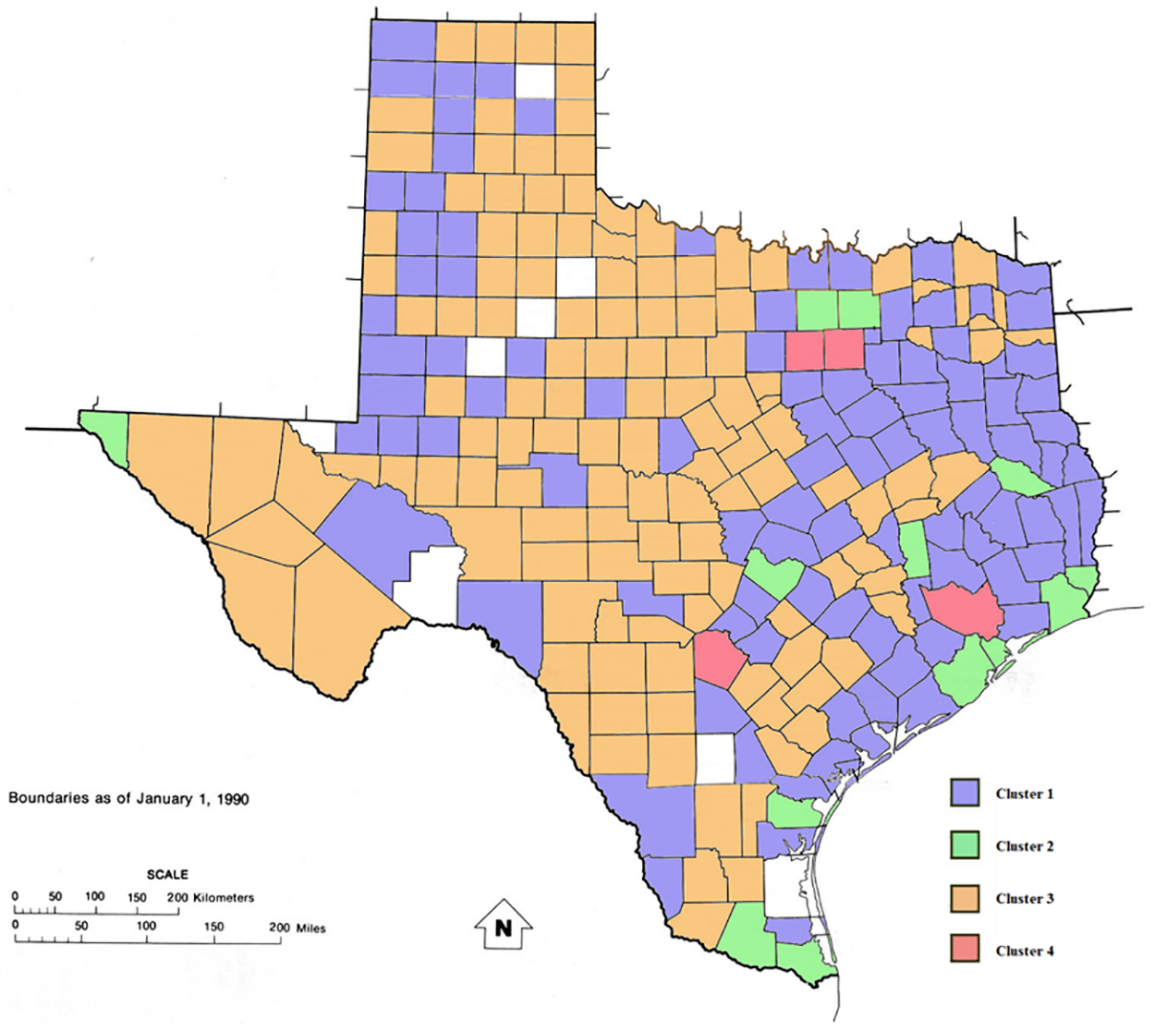
Map of current Texas counties with boundaries as of January 1, 1990 showing all 4 clusters: Cluster 1—blue; Cluster 2—green; Cluster 3—orange; Cluster 4 –red.

As stated above, when the number of cases is less than 16, both crude and age-adjusted rates, are not reported in the Texas cancer registry (suppressed data are indicated with the symbol ~). Actually, rates are not calculated due to instability in calculations. In such a circumstance only the population belonging to a specific county is available, while the number of cases has been censored if less than 6. This problem is particularly evident for counties belonging to cluster 1 and cluster 3. Actually, in the first group, 18 of the 100 counties register a total number of liver cancer cases, from 2002 to 2012, less than 16 while, for cluster 3, constituted by 129 counties, the number of such counties rises up to 89. Cluster 2 and 4 do not show any suppressed data. Even if the data was suppressed due to the low number of cases observed, it is relevant that this phenomenon is observed only in the clusters of counties with lower emission of pollutants (1 and 3). In Fig 4 is represented a map of current Texas counties with boundaries as of January 1, 1990 showing all 4 clusters: Cluster 1—blue; Cluster 2—green; Cluster 3—orange; Cluster 4 –red.

A rigorous comparison among liver cancer rates per clusters cannot be performed for two main reasons: 1) for cluster 1 and cluster 3 all data is not available (in particular, for cluster 3, 89 rates over 129 are suppressed); 2) population at risk in each cluster is different. The latter could emphasize the rates where population at risk is not numerous, leading to erroneous conclusions. In particular, for each cluster liver cancer rate distributions are determined with the non-parametric Kernel estimator and considerations supplied.

### Cluster 1

**Fig 5:**
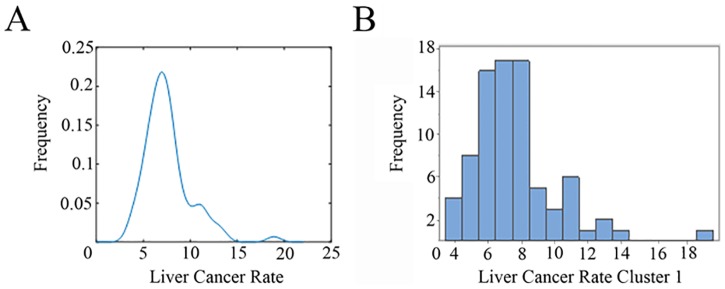
(A) Kernel estimator and (B) histogram for liver cancer rate in cluster 1.

In this cluster 18 counties out of 100 are suppressed. The mean population at risk in these counties in 11 years was 331,509 people, while population at risk for the remaining 82 counties, in the same period was 6,875,009 with a total number of cases of 5,551. Walker County (mean population of 65,874) exhibits the highest rate (18.9). The cluster rate is 7.34 in 100,000 people (Fig 5).

### Cluster 2

**Fig 6:**
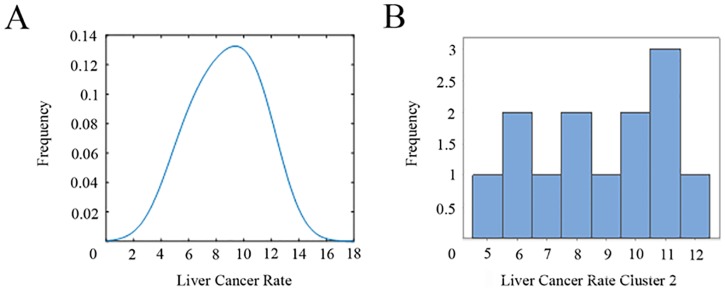
(A) Kernel estimator and (B) histogram for liver cancer rate in cluster 2.

Cluster 2 is constituted of 13 counties with a mean population of 5,470,124 people in the period 2002–2012. The incidence rate in this cluster is 7.62 in 100,000 population (Fig 6).

### Cluster 3

Cluster 3 is constituted of 129 counties with a mean population of 1,646,974 in the 11 years period. Among these, 5 counties show a rate of 0.0 while in 89 counties with population at risk of 678,694, data is suppressed. The total number of cases in the 11 years was 970 and the rate is 9.1 per 100,000. Brooks and Maverick counties present with the highest rates: 19.7% in 7,417 and 18.3% in 51,843 population respectively.

### Cluster 4

Cluster 4 is constituted by 4 counties with 9,502,282 population at risk. The total number of cases in 11 years is 8,752. The rate in 100,000 population is 8.37.

**Fig 7:**
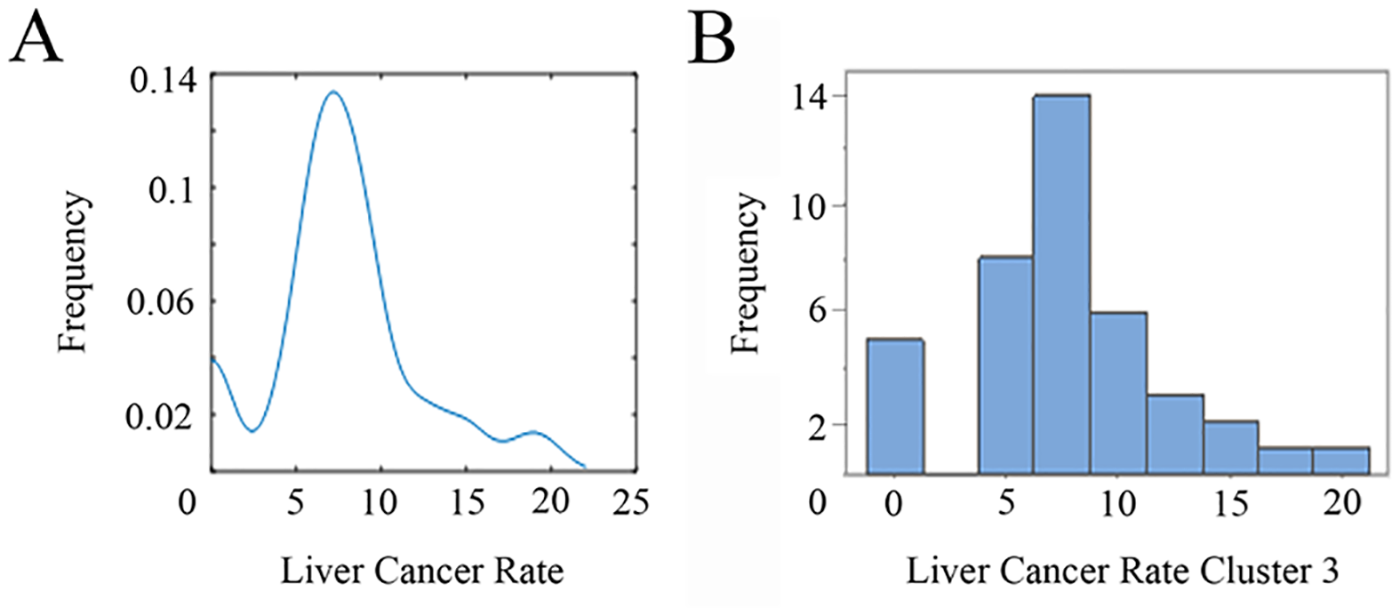
(A) Kernel estimator and (B) histogram for liver cancer rate in cluster 3.

**Fig 8:**
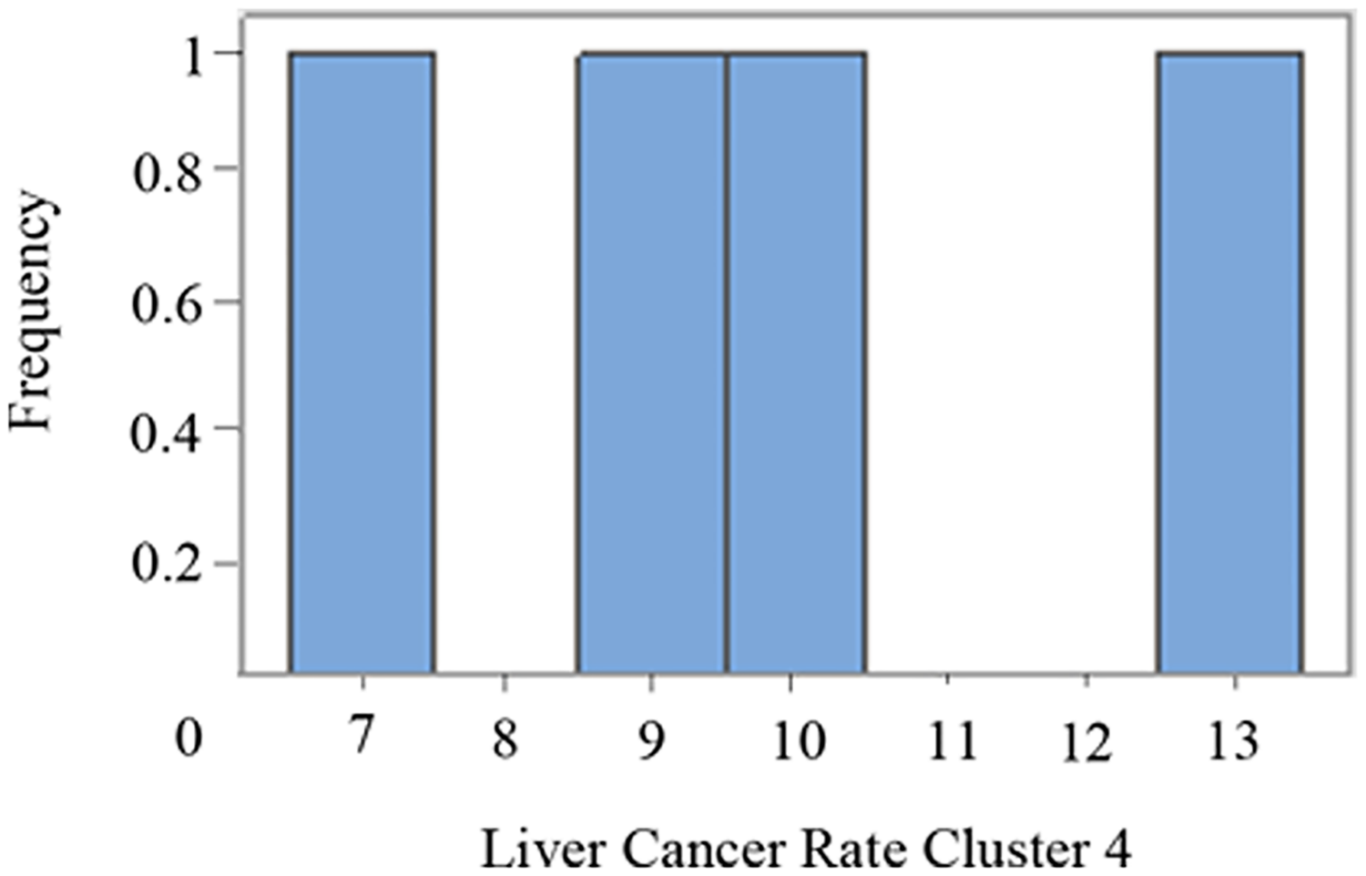
Histogram for liver cancer rate in cluster 4.

The histogram and the kernel estimator for the cluster liver cancer rate (age-adjusted) distribution shows cluster 3 (the least populated cluster) with the lowest content of pollutants. On the other hand, cluster 4 is more polluted and the most populated (Figs 7 and 8). Actually the 39.9% of the entire population of Texas lives in the four counties belonging to this last cluster.

Conclusions drawn on the basis of the observable data could lead to selection bias. In order to evaluate the impact of missing data on study results, Inverse Probability Weighting (IPW) was used. For cluster 1, the introduction of 18 missing values changes the cluster mean rate (per 100,000) from 7.34 to 7.49. Actually, the population at risk in these counties in the examined time window was 331,509 with respect to the population at risk in the 82 counties that was 6,875,009. For cluster 3, the mean cluster rate changes from 9.1 to 8.77. Actually the mean population at risk in these 89 counties in the period 2002–2012 was 678, 694, while the total mean population in the cluster was 1,646,974 as reported in Table 5.

**Table 5:** Mean population for each cluster in the period 2002–2012.

Cluster	Mean population per year
1	7,206,517
2	5,470,124
3	1,646,974
4	9,502,282

In Table 6 counties with the highest age-adjusted rate belonging to the different clusters are shown. In the last two columns the amplitude of the 95% CI on the mean age-adjusted rate and the mean population are reported for 2002–2012.

**Table 6:** Counties with the highest rate belonging to the four different clusters.

COUNTY(Cluster)	Rate age-adjusted	Amplitude of 95% CI	Mean Population
Brooks (3)	19.7	18.9	7417
Zavala (3)	15.4	15.2	11665
Jim Wells (3)	14.7	7.3	40599
Val Verde (1)	11.4	6.2	47610
Maverick (3)	18.3	8.2	51843
Starr (3)	12.5	6.3	59217
Walker (1)	18.9	6.6	65874
San Patricio (1)	12.6	5.4	66142
Webb (1)	13.5	3.6	234108
Jefferson (2)	9.7	2.3	249868
Galveston (2)	11.1	2.4	281674
Nueces (2)	11.9	2.3	331237
Cameron (2)	11.2	2.2	385833
El Paso (2)	10.8	1.5	759550
Bexar (4)	13	1.1	1612364
Dallas (4)	9.8	0.8	2314093
Harris (4)	9.4	0.7	3874433
STATE	8.5	0.3	23818182

## Future developments

In this study, Texas counties were divided into four groups according to the selection, through a Principal Component analysis model, of ten pollutants that account for the 94.9% of the variation in the original 253 pollutants dataset. Therefore, the subsequent discussion on the incidence rate distributions of primary liver cancer over the identified clusters was exclusively focused on the prioritized pollutants. This approach could hide a possible contribution of chemicals having a low-moderate variability on HCC. To overcome this limitation, a first step/result of a future research study is presented. Actually, through a stepwise Poisson regression model, other pollutants with a low variability have been identified. Results shows that Benzo[b]Fluoranthene, Benzo[b+k]Fluoranthene, 15-PAH, 2,4-Dichlorophenoxy Acetic Acid and Bis(2-Ethylhexyl)Phthalate constitute a set of significant regressors (p-value<0,0000) in the model with the ten prioritized ones. Table 7 shows the mean concentration of these pollutants in the four clusters. Even if the latters were identified only on the basis of the first set of the ten selected pollutants, it is interesting to show how their concentration is low (in tpy) with respect to the first ones (see Table 2). In Discussion their cancerogenic nature and use is presented.

**Table 7:** Mean pollutant concentration with low variability.

MEAN POLLUTANT (*tpy*) PRODUCTION/CLUSTERS				
Pollutants	CLUSTER 1	CLUSTER 2	CLUSTER 3	CLUSTER 4
Benzo[b]Fluoranthene	0,011502	0,011645	0,011562	0,011715
Benzo[b+k]Fluoranthene	1,985E-06	2,155E-06	2,010E-06	2,138E-06
15-PAH	0,193089	0,189423	0,191636	0,195213
2,4-Dichlorophenoxy Acetic Acid	0,075084	0,075859	0,075091	0,076234
Bis(2-Ethylhexyl)Phthalate	0,071885	0,080124	0,037863	0,040240

## Discussion

In our study, only 2,2,4-Trimethylpentane, Benzene, Ethyl Benzene, Formaldehyde, Hexane, Hydrochloric Acid, Methanol, Methyl Tert-Butyl Ether, Toluene, and Xylenes exhibited distinct loadings among all air pollutants studied. They contribute mostly to the *scores* of the PCA model, while for those pollutants with loadings close to 0 their contribution was statistically irrelevant. As shown in Table 1, the first PC was heavily loaded on 2,2,4-Trimethylpentane, Benzene, Ethyl Benzene, Formaldehyde, Methanol, Toluene, and Xylenes, while the second PC was heavily loaded on Hexane, Hydrochloric Acid, and Methyl Tert-Butyl Ether. The purpose of this study is to detect a possible “accordance” between the distribution of air environmental pollutants in the identified clusters and the mean incidences rates of liver cancer per cluster. The analysis does not intend to identify a cause-effect relationship between environmental pollutants and cancer rates. In fact liver cancer is a multifactorial disease and other possible causes were not considered in this study, however, the goal of this study is to identify possible *tuning* between the distribution of environmental pollutants in the 4 clusters and the cancer rates. Some of the pollutants selected are known carcinogens and others are not, in particular 2,2,4-Trimethylpentane is not recognized as liver carcinogenic compounds due to inadequate information even though it was observed in animal models to affect the action in hepatocytes metabolism with effect on liver weight [18] and mitogenic effects on hepatocytes [19–21]. Benzene and Ethyl Benzene have carcinogenic effects in the liver as was observed in mice experiments [22–24]. Ethyl benzene modulated enzymes and increased foci considered precursors of HCC neoplasia, [23, 25–28] but despite some evidence, it is not classified as a human carcinogen.

Formaldehyde (FA) is related with key events associated with tumorigenesis such as DNA reactivity, gene mutation, chromosomal breakages, aneuploidy, epigenetic effects, glutathione depletion, oxidative stress and cytotoxicity induced cellular proliferation [29, 30]. In a study [31], inhaled FA was found to cause DNA single-strand breaks in the liver of male rats. Evidence has shown that FA forms crosslinks in DNA and cellular proliferation increases considerably at concentrations > 6 ppm and amplifies the genotoxic effects of FA [32]. DNA damage was significantly induced in livers of rats by increasing FA concentration [31]. EPA considers FA to be a probable human carcinogen (cancer-causing agent) and has ranked it in EPA's Group B1.

No information is available on the carcinogenic effects of hexane in humans or animals and the EPA has classified hexane as a group D which is not classifiable as to human carcinogenicity, based on a lack of data concerning carcinogenicity in humans and animals [33, 34]. No relevant information is available on the carcinogenic effects of methanol and hydrochloric acid in humans or animals and the EPA has not classified methanol or hydrochloric acid with respect to carcinogenicity [35–37]. Methyl Tert-Butyl Ether (MTBE) showed its carcinogenic effects in mice liver but with conflicting results [38–40]. No recent information is available on the carcinogenic effects of MTBE in humans. Prolonged exposure to Toluene compounds may represent a risk factor for liver cancer [41]. The EPA states that workers exposed to toluene have reported limited or no evidence of the carcinogenicity potential of toluene. A limited amount of epidemiological studies have also failed to demonstrate increased risk of cancer from the inhalation of toluene. Finally, chronic inhalation in rats did not produce an increased incidence of treatment-related neoplastic lesions [42, 43].

Xylene is widely used in industry as a solvent and can be found in petroleum products. In a 2-year hospital-based case-control study conducted in northern Italy, it was found that xylene and toluene could have played a role in the development of liver cancer [41]. Both the International Agency for Research on Cancer (IARC) and EPA have found that there is insufficient information to determine whether or not xylene is carcinogenic and consider xylene not classifiable as to its human carcinogenicity.

Polycyclic aromatic hydrocarbons as 15-PAH, Benzo[b]Fluoranthene and Benzo[b+k]Fluoranthene are classified by the International Agency for Research on Cancer (IARC). In particular Benzo[b]Fluoranthene, Benzo[b+k]Fluoranthene and 2,4-Dichlorophenoxy Acetic Acid) and Bis(2-Ethylhexyl)Phthalate are in group 2B (possibly carcinogenic in human) [44–45].

In conclusion, in our study, we showed that selected relevant air pollutants produce a significant clustering of the Texan counties with respect to their concentration and discussed about the incidence rate distributions of liver cancer over the identified clusters.

A sort of association between the increased exposure to these pollutants and a higher presence of liver cancer in certain counties is suggested. However, considering the multifactorial nature of liver cancer, this study provides a new insight on this complex disease suggesting that environmental substances might play a role in the etiology of this cancer.

## Abbreviations

DA: Discriminant Analysis
EPA: Environmental Protection Agency
FA: Formaldehyde
HAP: Hazardous Air Pollutants
HBV: Hepatitis B Virus
HCC: Hepatocellular Carcinoma
HCV: Hepatitis C Virus
IARC: International Agency for Research on Cancer
MTBE: Methyl Tert-Butyl Ether
NATA: National Scale Air toxic Assessment
NIAAA: National Institute on Alcohol Abuse and Alcoholism
PCA: Principal Component Analysis
PHA: Polycyclic aromatic hydrocarbons
TCR: Texas Cancer Registry
